# Screening practices for antimicrobial-resistant organisms in a network of Canadian acute care hospitals

**DOI:** 10.1017/ash.2024.385

**Published:** 2025-06-30

**Authors:** Andrew Neitzel, Jessica J. Bartoszko, Erin McGill, Maureen Buchanan-Chell, Jenine Leal, Robyn Mitchell, Stephanie Smith, Reena Titoria, Oliva Varsaneux, Charles Frenette

**Affiliations:** 1 Public Health Agency of Canada, Ottawa, ON, Canada; 2 Alberta Health Services, Edmonton, AB, Canada; 3 Provincial Health Services Authority, Vancouver, BC, Canada; 4 McGill University Health Centre, Montreal, QC, Canada

## Abstract

This study investigates screening practices for antimicrobial-resistant organisms (AROs) in seventy-five hospitals participating in the Canadian Nosocomial Infection Surveillance Program (CNISP). Screening practices varied with widespread MRSA screening, selective carbapenemase-producing organisms (CPO) screening, and limited vancomycin-resistant *Enterococcus* (VRE) screening. These findings may help interpret ARO rates within CNISP hospitals and inform screening practices.

## Introduction

The increasing global prevalence of antimicrobial-resistant organisms (AROs) poses a significant and ongoing challenge to human health, with patients in hospital settings being particularly vulnerable.^1^ Effectively addressing antimicrobial resistance relies in part on the implementation of robust screening practices. Screening helps to identify patients who are either colonized or infected with AROs and facilitate the implementation of essential control measures, such as contact precautions, to minimize the risk of transmission to other patients.^2,3^

Screening practices across Canadian hospitals may differ according to provincial or territorial policies, local epidemiological factors, evolving guidelines, and varying levels of implementation^4,5^ in practices, which may affect comparability of rates of ARO infections.^6^

To better understand variations in ARO incidence in Canada, we aim to describe the different screening practices of acute care hospitals within the Canadian Nosocomial Infection Surveillance Program (CNISP) network for the 2022 calendar year.

## Methods

### Data collection

CNISP is a collaboration between the Public Health Agency of Canada (PHAC), including the National Microbiology Laboratory, the Association of Medical Microbiology and Infectious Disease Canada, and sentinel hospitals across Canada. CNISP is voluntary, funded by PHAC, and gathers routine epidemiological, clinical, microbiological, and molecular data for patients with select healthcare-associated infections (HAIs) and AROs. In 2022, CNISP consisted of 88 hospitals in ten provinces and one territory. This study utilized data collected via the Enhanced Hospital Profile (EHP)—an expert-reviewed and standardized questionnaire—that collects hospital-level data annually related to select ARO screening practices.^7^ The AROs included in this questionnaire consist of methicillin-resistant *Staphylococcus aureus* (MRSA), vancomycin-resistant *Enterococcus* (VRE), and carbapenemase-producing organisms (CPOs). For each ARO, we collected data on whether screening was conducted in all patients on admission, high-risk patients on admission, patients transferred from another healthcare facility and/or patients during hospitalization. For hospitals that reported screening high-risk patients on admission, we collected additional information on which hospitals screened transplant, hematology/oncology, and dialysis patients. Hospitals that reported screening patients during hospitalization were requested to provide details on the specific units included in this prevalence screening process. For CPO, additional questions were asked regarding screening practices for domestic and international hospitalizations.

For the analysis, e defined screening as the systematic identification of patients susceptible to colonization or infection by AROs. In cases where risk factors are identified, the process extends to obtaining suitable specimens for further assessment.^7^ We defined universal admission screening as encompassing all patients upon admission, while targeted screening pertained to high-risk patients upon admission, patients during hospitalization, and those transferred from another healthcare facility.^7^

We grouped Canadian provinces and territories into regions based on geographical proximity. The eastern region comprised New Brunswick, Nova Scotia, Prince Edward Island, Newfoundland, and Labrador. The central region included Ontario and Quebec. The western region comprised British Columbia, Alberta, Saskatchewan, and Manitoba. The Northern region included Nunavut.

## Data analysis

The primary unit of analysis for our descriptive study was the hospital. Hospitals that reported data on MRSA, VRE, and CPO screening practices were eligible for analysis. Using the number of completed responses for each question, we reported proportions and percentages for categorical data and medians (interquartile ranges) for continuous data that were not normally distributed. Our analysis focused on 2022 data as it was the most recent and complete data. All analyses were conducted using R statistical software (R version 4.2.1).^8^

## Results

In the 2022 surveillance year, 75 hospitals representing 85% (75/88) of CNISP participating hospitals completed the EHP. All 75 hospitals submitted data for CPO, MRSA, and VRE screening. Hospitals were distributed across western (28/75 (37%)), eastern (26/75 (35%)), central (20/75 (27%)), and northern (1/75 (1%)) Canada. Hospital types included adult (42/75 (56%)), mixed adult and pediatric (21/75 (28%)), and pediatric (12/75 (16%)) hospitals. The median number of hospital beds was 248 (IQR = 124,461). Over two-thirds were teaching hospitals (51/75 (68%)).

Screening was most common for MRSA (73/75 (97%)) and CPO (70/75 (93%)) and less common for VRE (52/75 (69%)). Among the hospitals that screened for MRSA, 50 hospitals screened high-risk patients on admission (50/73 (69%)), 47 screened patients during hospitalization (47/73 (64%)), and 45 screened patients previously hospitalized in the last 12 months (45/73 (61%)). Among the hospitals that screened for VRE, 42 hospitals screened high-risk patients on admission (42/52 (81%)), 38 screened patients during hospitalization (38/52 (73%)), and 30 screened patients previously hospitalized in the last 12 months (30/52 (58%)). Among the hospitals that screened for CPO, 66 hospitals screened high-risk patients on admission (66/70 (94%)), 44 screened patients during hospitalization (44/70 (63%)), and 63 screened patients previously hospitalized in the last 12 months (63/70 (90%)). CPO screening for patients with a previous hospital admission outside of Canada in the last 12 months (54/70 (77%)) was more common than for patients with a previous Canadian hospital admission (33/70 (47%)).

**Figure 1.**
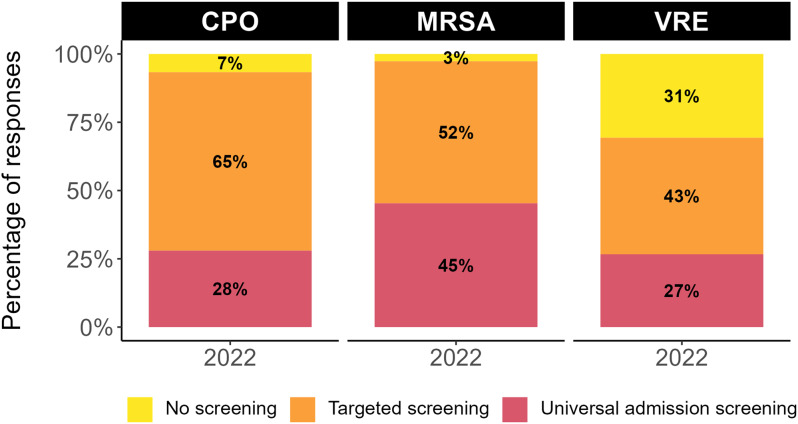
Proportion of the type of screening practices (universal, targeted, and no screening) for CPO, MRSA, and VRE within the CNISP hospital network. Acronyms: methicillin-resistant *Staphylococcus aureus* (MRSA), vancomycin-resistant *Enterococcus* (VRE), and carbapenemase-producing organisms (CPOs).

Among the different types of screening practices, targeted screening was the most common. Universal admission screening was most frequently reported for MRSA (34/75, 45%) compared to CPO (21/75 (28%)) and VRE (20/75 (27%)). Screening was least common for VRE, with one-third of hospitals not screening (23/75, 31%) (Figure 1).

Across the AROs of interest, the percentage of hospitals that screened select high-risk subgroups and ward inpatients ranged from 19% to 73% (Table 1 **)**. Admission screening was most common among hematology/oncology patients for MRSA (27/37 (73%)), VRE (17/36 (47%)), and CPO (11/28 (39%)) (Table 1). Similarly, hematology/oncology patients were most commonly screened during hospitalization for MRSA (17/37 (46%)), VRE (24/36 (67%)), and CPO (14/28 (50%)) (Table 1).

**Table 1.** The distribution of screening practices among high-risk patients and patients during hospitalization by organism

Number of screening hospitals per organism^a^	MRSA	VRE	CPO
High-risk patients on admission			
Transplant patients	16/27 (59%)	7/18 (39%)	10/25 (40%)
Hematology/oncology patients	27/37 (73%)	11/28 (39%)	17/36 (47%)
Dialysis patients	24/43 (56%)	6/31 (19%)	10/41 (24%)
Patients during hospitalization			
Transplant patients	12/27 (44%)	8/18 (44%)	14/25 (56%)
Hematology/oncology patients	17/37 (46%)	14/28 (50%)	24/36 (67%)
Dialysis patients	14/43 (33%)	7/31 (23%)	20/41 (49%)
Acute medical ward patients	15/63 (24%)	13/44 (30%)	16/61 (26%)
General surgical ward patients	16/58 (28%)	9/39 (23%)	15/56 (27%)
Specialized surgical ward patients	14/31 (45%)	5/20 (25%)	11/28 (39%)

Note. Denominators differ due to stratification by both screening for organism and hospital services provided.

a Sites that do not screen for methicillin-resistant Staphylococcus aureus (MRSA), vancomycin-resistant Enterococcus (VRE), and carbapenemase-producing organisms (CPOs) were excluded from this table.

## Discussion

Among this subset of Canadian hospitals, universal admission screening was found to be the most common for MRSA (34/75 (45%)), with a very small percentage of hospitals not doing any screening (2/75 (3%)). For CPO, nearly two-thirds of hospitals conduct targeted screening (49/75 (65%)) compared to universal screening approaches (21/75 (28%)). VRE was screened for the least, with over one-third of hospitals not conducting any screening (23/75 (31%)). Since 2012, several Canadian provinces and territories discontinued admission screening and contact precautions for VRE.^9^

The findings that CPO screening for patients with a previous hospital admission outside of Canada in the last 12 months (54/70 (77%)) was higher than for those with a previous Canadian hospital admission in the last 12 months (33/70 (47%)) is particularly noteworthy. Rates of CPO infection acquired within Canadian hospitals have been increasing in Canada since 2015;^10^ however, screening practices still prioritize international hospitalizations over domestic. This discrepancy highlights a potential opportunity for additional CPO screening.

Strengths of this study include its representative sample of Canadian acute care hospitals, which included geographical representation from all ten provinces and one territory, and use of standardized definitions. However, while hospitals self-report screening practices, the actual utilization in practice may vary, introducing a potential discrepancy between reported and implemented measures. To address a data collection limitation, we assumed respondents answered all questions that applied to them and interpreted a lack of response as a negative response (e.g., no MRSA screening).

Quantifying the variability in ARO screening practices helps contextualize ARO infection rates within CNISP hospitals. These data may identify opportunities for improved infection prevention and control measures, including improvements to screening practices. Future research may explore differences in regional screening policies, the relationship between ARO screening practices and rates of AROs, contributing to ongoing efforts to improve infection control measures within hospital settings.
